# Who is your community? Co-designing a community engagement strategy for an emerging data platform.

**DOI:** 10.23889/ijpds.v7i3.2107

**Published:** 2022-08-25

**Authors:** Kim Naude, Emily Parker, Roisin McNaney, Michelle Daniel, Richard Beare, Velandai Srikanth, Nadine Andrew

**Affiliations:** 1 National Centre for Healthy Ageing; 2 Monash University; 3 Peninsula Health

## Objectives

To co-design with older Australian consumers and stakeholders a strategy for engaging the wider community with an emerging data platform, involving linked routinely collected electronic health data for research. To develop strategies to address consumer concerns of data use, privacy and safety.

## Approach

The National Centre for Healthy Ageing (NCHA) Data Platform aims to make better use of existing health data to lead transformative research. Facilitated workshops were conducted with consumer representatives in March 2022. Use case scenarios were created following interviews with NCHA researchers; these were used to facilitate discussion around benefits and challenges of health data related research. Predetermined questions were given to two participant groups to develop strategies to engage the broader community with the Data Platform; ideas were presented to the whole group to prompt further discussion. Workshops were audio-recorded; transcripts are being analysed using thematic data analysis.

## Results

Two workshops were conducted (workshop 1=7 participants; workshop 2=9 participants). Participants were >65 years, of mixed gender (8 women; 8 men), used the same health service, and resided in the Frankston/Mornington Peninsula region of Victoria, Australia. Preliminary findings suggest an essential element to the design of an effective community engagement strategy with a data platform is the understanding of who the target community is (e.g. age, culture, and health/digital literacy). Conveyed information needs to be simple, clear and devoid of academic and/or technical jargon. Community engagement strategies should respect consumers’ genuine fear of who can access their data and concerns around whether their data is identifiable. Overall, participants expressed optimism in the NCHA Data Platform and its ability to directly impact their well-being.

## Conclusion

Preliminary findings from this study have identified effective strategies to engage the older community with an electronic health data platform, an often complex and technical concept. The co-design of a consumer engagement strategy allows the NCHA to broaden its network and ensure research outputs meet the needs of the older community.

